# Cases report: severe colonic bleeding in ulcerative colitis is refractory to selective transcatheter arterial embolization

**DOI:** 10.1186/s12876-019-0970-8

**Published:** 2019-04-16

**Authors:** Jose Miranda-Bautista, Lucía Diéguez, Gracia Rodríguez-Rosales, Ignacio Marín-Jiménez, Luis Menchén

**Affiliations:** 1 Servicio de Aparato Digestivo. Hospital General Universitario Gregorio Marañón, Instituto de Investigación Sanitaria Gregorio Marañón, C/ Dr. Esquerdo 46, 28007 Madrid, Spain; 2 Servicio de Radiología. Hospital General Universitario Gregorio Marañón, Instituto de Investigación Sanitaria Gregorio Marañón, Madrid, Spain; 3 Departamento de Medicina, Universidad Complutense de Madrid, Madrid, Spain; 4 Centro de Investigación Biomédica en Red de Enfermedades Hepáticas y Digestivas (CIBEREHD), Madrid, Spain

**Keywords:** Ulcerative colitis, Bleeding, Selective transcatheter arterial embolization, Colectomy, Case report

## Abstract

**Background:**

Severe haemorrhage is an uncommon but life-threatening complication of ulcerative colitis (UC). Superselective transcatheter embolization has shown to be an effective and safe therapeutic modality in patients with lower gastrointestinal bleeding of various aetiologies; nevertheless, its role in UC-related acute bleeding is unknown.

**Cases presentation:**

Efficacy and safety of selective transcatheter arterial embolization in three consecutive UC patients diagnosed with massive haemorrhage admitted in a tertiary institution are reported. In all patients computed tomography scan showed active arterial haemorrhage from ascendant or sigmoid colon; subsequent arteriography demonstrated active arterial bleeding from colic branches of the superior or inferior mesenteric arteries, and selective transcatheter embolization was performed with immediate technical success in all three cases. Nevertheless, rebleeding requiring subtotal colectomy occurred between 5 h and 6 days after the procedure.

**Conclusions:**

Transcatheter arterial embolization is not an effective therapeutic approach in UC patients with severe, acute colonic haemorrhage. Colectomy should not be delayed in this setting.

## Background

Ulcerative colitis (UC) is a chronic inflammatory condition affecting the mucosal layer of the colon. Up to 3% of UC patients, mainly those with pancolonic disease and in the setting of an acute flare [1, 2], may develop acute severe lower gastrointestinal bleeding – usually attributed to diffuse mucosal ulceration – during the course of their disease. According to guidelines, it represents an indication of urgent colectomy [3, 4]; indeed, this life-threatening complication accounts for up to 10% of all urgent colectomies for UC [5]. However, morbidity and even mortality following urgent colectomy in this setting are not negligible [5]. Improvements in interventional radiology techniques treating lower gastrointestinal bleeding have increased the expectancy of conservative treatments in this complication. In this sense, several studies show excellent results concerning technical and clinical success of superselective transcatheter embolization in lower gastrointestinal bleeding, avoiding surgery in more than two-thirds of patients [6–8]. However, patients with inflammatory bowel disease (IBD) are generally excluded from these series; to our knowledge, only one case of a patient diagnosed with long-standing UC and a massive haemorrhage successfully treated with transcatheter arterial embolization has been published up to date [9]. However, the authors could not demonstrate that the colonic haemorrhage was due to a UC exacerbation, as colonoscopy did not show any mucosal ulceration. Hereby, we present a single centre experience of all three cases of massive haemorrhage in UC patients treated with transcatheter arterial embolization since 2011. Written informed consent was obtained from all patients.

### Case reports

#### Case report #1, January 2011

A 27-year-old Caucasian woman was admitted to the Emergency Department of our Institution because of bloody diarrhoea – up to 10 bowel movements per day – during the last month, 3 weeks after quitting smoking. Physical examination showed no abnormalities but confirmed haematochezia on digital rectal examination. Colonoscopy showed continuous severe colonic inflammation with small ulcers from the anus to the descendent colon, classified as grade 3 in Mayo endoscopic sub-score and 3 points in Ulcerative Colitis Endoscopic Index of Severity (UCEIS); complete examination was not performed because of the risk of perforation. Empirical antibiotic treatment with ciprofloxacin and metronidazole, as well as oral and rectal mesalamine were started and partial symptomatic improvement was achieved. Venous thrombosis prophylaxis with subcutaneous enoxaparin, 40 mg per day, was started. At admittance, haemoglobin, white cell count, platelets, fibrinogen and C reactive protein (CRP) were within the reference range. Stool cultures were negative. Cytomegalovirus (CMV) infection was also ruled out in colonic biopsies (polymerase chain reaction – PCR – and, later, immunohistochemistry). As bloody diarrhoea persisted 48 h later, and histopathological examination of colonic biopsies showed crypt distortion, a mixed inflammatory infiltrate of the *lamina propria* and crypt abscesses suggesting the diagnosis of UC, intravenous methylprednisolone (1 mg per kg of weight, daily) was started. After 3 days of corticosteroids the patient achieved partial clinical response (6 bowel movements per day, Edinburgh index 2 points, CRP within the normal range); nevertheless, 2 weeks later infliximab therapy (5 mg/kg of weight) was started due to sustained clinical activity, with 10 bloody bowel movements per day and a progressive increase of CRP levels, up to 10 mg/dL. Three days after the first dose of infliximab, the patient presented a massive lower bleeding with haemodynamic instability and severe anaemia; CT scan showed active arterial haemorrhage from ascendant colon; a subsequent arteriography demonstrated active arterial bleeding from a colic branch of the superior mesenteric artery; selective transcatheter embolization with platinum microcoils (MicroNester©, Cook Medical) was performed with immediate technical success; nevertheless, the patient persisted with rectal bleeding 2 days after embolization, requiring laparoscopic subtotal colectomy and ileostomy. Pathological evaluation of the colon confirmed the diagnosis of UC. Eight days after surgery the patient was discharged.

#### Case report #2, December 2016

A 39-year-old Caucasian women diagnosed with pancolonic UC 3 months earlier was admitted into our department because of a severe flare of the disease and an associated *Clostridium difficile* infection. She had started treatment with 160 mg of adalimumab due to steroid-dependent disease 9 days before, after performing a rectosigmoidoscopy which showed erythema, lack of vascular pattern, friability, erosions and aphtae (grade 2 of Mayo endoscopic sub-score, 3 points in the UCEIS index); complete examination was not performed because of the risk of perforation. CMV infection was ruled out in rectal biopsies. Laboratory findings showed haemoglobin of 12.3 g/dL, 14.500 leucocytes per mL, fibrinogen of 873 mg/dL, serum creatinine of 1.18 mg/dL, and CRP of 9.2 mg/dL. Metronidazole was started and a second dose of adalimumab was administered. Venous thrombosis prophylaxis with subcutaneous enoxaparin, 40 mg per day, was started. As fever, bloody diarrhoea and abdominal pain persisted, intravenous corticosteroids at 1 mg per kg of weight dosage were started; the patient was also switched to intravenous infliximab at 10 mg per kg of weight. CMV PCR in blood samples was negative, and faecal samples ruled out *Clostridium difficile* persistence. After 3 days partial response was observed – 3 bloody stools per day, no colonic dilation on abdominal X-ray (Edinburgh index 0 points), CRP 5.6 mg/dL – and a second infliximab infusion at 10 mg per kg of weight was administered 4 days later. After 12 h she presented massive lower haemorrhage, hypotension, tachycardia and severe anaemia needing supportive intervention with intravenous fluids and packed red blood cells transfusion. A CT scan showed active arterial bleeding in the ascendant colon; subsequent arteriography showed active arterial bleeding from a colic branch of the superior mesenteric artery that could be embolized with polyvinyl alcohol particles (Contour TM, Boston Scientific), with immediate technical success. Although clinical situation became steady, 6 days later she developed a massive haemorrhage; finally, urgent laparoscopic subtotal colectomy and terminal ileostomy were done. Clinical evolution was favourable, and the patient was discharged with corticosteroid tapering. Pathological evaluation of the colon confirmed the diagnosis of UC without infectious complications or dysplasia.

#### Case report #3, January 2018

A 42-year-old Hispanic woman with history of autoimmune hypothyroidism and uncomplicated Caesarean section 6 weeks before, was admitted to our hospital because of fever, abdominal pain and bloody diarrhoea – confirmed after digital rectal examination – with up to 8 bloody bowel movements per day for the last 4 days. Physical examination also showed diffuse abdominal tenderness without signs of peritonitis. Laboratory exam showed haemoglobin of 9.2 g/dL, leucocytes 11.610 per mL, fibrinogen of 828 mg/dL, CRP of 4.4 mg/dL and ALT of 127 UI/mL, with no other significant alterations. A CT-scan ruled out obstetric complications, showing marked oedema of rectum and sigmoid colon; rectosigmoidoscopy showed severe inflammation (grade 3 in Mayo endoscopic sub-score; 5 points in UCEIS index) with deep and large ulcers affecting the whole circumference from rectum to 45 cm from anus, erythema and erosions; complete examination was not performed because of the risk of perforation. Despite negative stool cultures, ciprofloxacin and metronidazole was initiated, as well as oral (4 g per day) and topical mesalamine (1 g per day), with favourable response. Histopathological examination of colonic biopsy specimens showed crypt distortion, a mixed inflammatory infiltrate of the *lamina propria* and crypt abscesses, suggesting UC. After 6 days of therapy, she presented a lower massive gastrointestinal bleeding, with haemodynamic instability and severe anaemia needing supportive intervention with intravenous fluids and packed red blood cells transfusion. Corticosteroids at 1 mg per kg of weight dosage were started. Urgent CT-scan showed active arterial bleeding in the sigmoid colon; subsequent arteriography performed 16 h from the beginning of symptoms showed active bleeding from a branch of the inferior mesenteric artery. After selective catheterization, embolization with polyvinyl alcohol particles (Contour TM, Boston Scientific) was performed with immediate technical success, as demonstrated by complete occlusion of the bleeding vessel confirmed by a control radiographic series with the injection of contrast in the inferior mesenteric artery. However, 5 h after the procedure she restarted with persistent bleeding, abdominal pain, need for additional packed red blood cells transfusion and, finally, urgent open subtotal colectomy and terminal ileostomy was performed. Pathological evaluation of the colon showed signs of UC without infectious complication or dysplasia.

## Discussion and conclusions

Severe haemorrhage is an uncommon but life-threatening complication of UC, representing 0.1 to 1.4% of hospital admissions for this disease [1, 2]. It mainly occurs in patients with extensive colitis, in the setting of an acute flare of the disease [1, 2] – with a relatively high rate of concomitant toxic megacolon – and should be considered as an indication for immediate total proctocolectomy and ileostomy or subtotal colectomy, since the presence of an endoscopically treatable lesion is exceptional [2]. Furthermore, and as in the three cases reported herein, it has been reported that acute severe bleeding is frequently an early complication during the course of UC [1].

Current guidelines concerning UC management recommend as an absolute indication for emergency surgery the presence of massive haemorrhage despite maximal medical therapy [3, 10]. Urgent subtotal colectomy with preservation of the rectum is the technique of choice, but this procedure may require experienced surgeons that ideally can perform the resection by laparoscopy. On the other hand, bleeding often occurs in patients in the early phase of the disease or even in patients, in whom UC diagnosis is not confirmed, leading to difficulties of accepting the radical resection and a stoma. These issues make urgent decisions even more difficult, and the patient or their physicians may attempt a more conservative management. In addition, morbidity and mortality of urgent colectomy, although decreasing in the last decades, are not negligible. A systematic review of colectomy in acute colitis [5] (mainly UC) reports morbidity of 40.1%, predominantly because of wound infection-dehiscence, intraabdominal abscess or small bowel obstruction; haemorrhage was the indication of urgent colectomy in only 7.7% of the patients studied. Finally, interventional radiology is achieving increasing technical success in treating lower gastrointestinal bleeding and avoiding surgery. Although the risk of colonic infarction after embolization is higher than in gastric and small bowel bleeding – because of the lack of an overlapping arterial blood supply in the colonic vasculature – the development of selective microcatheters and new embolization agents, such as microcoils and polyvinyl alcohol particles, as in these three cases reported herein, allows embolization of smaller vessels, reducing the risk of colonic infarction [6]. Thus, selective transcatheter embolization represents a safe, effective and widely employed alternative to surgery in endoscopically refractory lower gastrointestinal bleeding [6–8]. Management of this potentially life-threatening complication was decided in agreement with gastroenterologists, surgeons, radiologists, and patients. In our cases, the doubts concerning radical surgery as the only feasible therapy were expressed by the team in charge, probably because of scarce experience of surgical team performing a colectomy in non-regular working time, a single site of bleeding identified and radiological expert availability, and finally embolization was considered after discussing with the patient.

To our knowledge, there is only one report of an effective selective transcatheter embolization in a UC patient [9]; it is tempted to speculate, therefore, that this fact may suggest a publication bias. However, the authors could not demonstrate that the colonic haemorrhage was due to a UC exacerbation, as colonoscopy, limited by the fresh blood that filled the lumen, did not show any mucosal ulceration. In contrast, the cases herein reported were hospitalised because a UC flare with current or recent endoscopic evaluation confirming mucosal damage, finding a Mayo endoscopic sub-score of 2 or more.

Some reports have shown effective endoscopic treatment with clips [11, 12] or injection of absolute ethanol and 1% polydocanol [13] applied on an active site of bleeding in the colon of these patients. In our cases, the severity of bleeding, the need of bowel cleansing in a life-threatening emergency and the thought that the blood loading the large bowel would hamper the visualization of the site of bleeding and its treatment made us choose endovascular treatment.

Massive lower haemorrhage in patients diagnosed with Crohn’s disease is less frequent than in UC, and the treatment of this complication in Crohn’s disease is controversial. Surgery remains the salvage treatment, but endoscopic and radiologic techniques may be an option in expertise hospitals [14]. Furthermore, another study from Spain showed effective transcatheter arterial embolization in two Crohn’s disease patients with colonic severe bleeding [15].

The three cases reported herein show our experience in the conservative treatment of severe bleeding in UC patients with selective transcatheter embolization and, despite initial technical success, all of them required surgical treatment within few days after the procedures. All of them were performed by the same experienced interventional radiologist (GRR). Reasons for unsuccessful transcatheter arterial embolization in this setting may include both technical failures and the development of complications such as bowel ischaemia [6–8]. The main cause for technical failure of transcatheter arterial embolization described in the literature is the inability to perform superselective catheterization of the bleeding vessels; in our series, technical success was accomplished in all three cases, as demonstrated by complete occlusion of the bleeding vessels confirmed by a control radiographic series. On the other hand, it is known that superselective embolization reduces the frequency of secondary colonic ischaemia to less than 13% [6–8], and none of our patients developed this complication. Clinical or anatomical factors related to rebleeding after colonic arterial embolization have not been clearly established, although the underlying diagnosis seems to be relevant. Relatively early rebleeding observed in these three cases seems to be related to the intrinsic characteristics of a disease affecting the complete colon with marked mucosal hyperaemia and frequent deep ulcers.

In summary, and taking into account the limitations of the present case series, there is no evidence that selective transcatheter arterial embolization represents an effective therapeutic alternative in UC patients with severe, acute colonic bleeding. Even in a tertiary care centre with wide expertise in endovascular procedures, delaying surgery in this setting seems to be futile and to imply a loss of functional reserve that may have deleterious consequences in these patients. Consequently, urgent colectomy is still the most effective and reliable therapy choice for massive haemorrhage in UC.

## Abbreviations

CMV: Cytomegalovirus
CRP: C reactive protein
CT: Computed tomography
IBD: Inflammatory bowel disease
UC: Ulcerative colitis
